# *DBP* rs16846876 and rs12512631 polymorphisms are associated with progression to AIDS naïve HIV-infected patients: a retrospective study

**DOI:** 10.1186/s12929-019-0577-y

**Published:** 2019-10-23

**Authors:** María Ángeles JIMÉNEZ-SOUSA, José Luis JIMÉNEZ, Amanda FERNÁNDEZ-RODRÍGUEZ, José María BELLÓN, Carmen RODRÍGUEZ, Melchor RIERA, Joaquín PORTILLA, Ángeles CASTRO, María Ángeles MUÑOZ-FERNÁNDEZ, Salvador RESINO

**Affiliations:** 1 Unidad de Infección Viral e Inmunidad, Centro Nacional de Microbiología, Instituto de Salud Carlos III, Carretera Majadahonda- Pozuelo, Km 2.2, 28220 Majadahonda, Madrid, Spain; 2 Plataforma de Laboratorio, Hospital General Universitario “Gregorio Marañón”, Madrid, Spain; 3 Fundación para la Investigación Biomédica, Hospital General Universitario Gregorio Marañón, Instituto de Investigación Sanitaria Gregorio Marañón (IiSGM), Madrid, Spain; 4 Centro Sanitario Sandoval, Instituto de Investigación Sanitaria San Carlos (IdISSC), Madrid, Spain; 5 Servicio de Medicina Interna-Infecciosas, Hospital Universitario “Son Espases”, Palma de Mallorca, Spain; 6 Servicio de Enfermedades Infecciosas, Hospital General Universitario de Alicante, Alicante, Spain; 7 Unidad de Enfermedades Infecciosas, Servicio de Medicina Interna, Complejo Hospitalario Universitario a Coruña (CHUAC), A Coruña, Spain; 8 Sección Inmunología, Laboratorio InmunoBiología Molecular, Hospital General Universitario Gregorio Marañón, IiSGM, and Spanish HIV HGM BioBank, Madrid, Spain; 9 Networking Research Center on Bioengineering, Biomaterials and Nanomedicine (CIBER-BBN), Madrid, Spain

**Keywords:** Single nucleotide polymorphisms, *DBP*, LTNPs, AIDS, Non-progression

## Abstract

**Background:**

Most of the circulating Vitamin D (VitD) is transported bound to vitamin D-binding protein (DBP), and several DBP single nucleotide polymorphisms (SNPs) have been related to circulating VitD concentration and disease. In this study, we evaluated the association among *DBP* SNPs and AIDS progression in antiretroviral treatment (ART)-naïve-HIV-infected patients.

**Methods:**

We performed a retrospective study in 667 patients who were classified according to their pattern of AIDS progression (183 long-term non-progressors (LTNPs), 334 moderate progressors (MPs), and 150 rapid progressors (RPs)) and 113 healthy blood donors (HIV, HCV, and HBV negative subjects). We genotyped seven *DBP* SNPs (rs16846876, rs12512631, rs2070741, rs2282679, rs7041, rs1155563, rs2298849) using Agena Bioscience’s MassARRAY platform. The genetic association was evaluated by Generalized Linear Models adjusted by age at the moment of HIV diagnosis, gender, risk group, and *VDR* rs2228570 SNP. Multiple testing correction was performed by the false discovery rate (Benjamini and Hochberg procedure; q-value).

**Results:**

All SNPs were in HWE (*p* > 0.05) and had similar genotypic frequencies for *DBP* SNPs in healthy-controls and HIV-infected patients. In unadjusted GLMs, we only found significant association with AIDS progression in rs16846876 and rs12512631 SNPs. In adjusted GLMs, *DBP* rs16846876 SNP showed significant association under the recessive inheritance model [LTNPs vs. RPs (adjusted odds ratio (aOR) = 3.53; *q*-value = 0.044) and LTNPs vs. MPs (aOR = 3.28; *q*-value = 0.030)] and codominant [LTNPs vs. RPs (aOR = 4.92; *q*-value = 0.030) and LTNPs vs. MPs (aOR = 3.15; *q*-value = 0.030)]. Also, we found *DBP* rs12512631 SNP showed significant association in the inheritance model dominant [LTNPs vs. RPs (aOR = 0.49; *q*-value = 0.031) and LTNPs vs. MPs (aOR = 0.6; *q*-value = 0.047)], additive [LTNPs vs. RPs (aOR = 0.61; *q*-value = 0.031)], overdominant [LTNPs vs. MPs (aOR = 0.55; *q*-value = 0.032)], and codominant [LTNPs vs. RPs (aOR = 0.52; *q*-value = 0.036) and LTNPs vs. MPs (aOR = 0.55; *q*-value = 0.032)]. Additionally, we found a significant association between *DBP* haplotypes (composed by rs16846876 and rs12512631) and AIDS progression (LTNPs vs RPs): *DBP* haplotype AC (aOR = 0.63; *q*-value = 0.028) and the *DBP* haplotype TT (aOR = 1.64; *q*-value = 0.028).

**Conclusions:**

*DBP* rs16846876 and rs12512631 SNPs are related to the patterns of clinical AIDS progression (LTNP, MP, and RP) in ART-naïve HIV-infected patients. Our findings provide new knowledge about AIDS progression that may be relevant to understanding the pathogenesis of HIV infection.

## Background

In the absence of antiretroviral treatment (ART), human immunodeficiency virus (HIV)-infected patients have a highly variable progression to acquired immunodeficiency syndrome (AIDS) [1]. Most HIV-infected patients slowly progress towards AIDS during an extended period after HIV seroconversion (moderate progressors, MPs). As opposed, extreme phenotypes regarding the virological and clinic-immunological range of HIV disease have been described [2]. Whereas a subgroup of HIV-infected patients (long-term non-progressors, LTNPs) do not progress to AIDS (absence of clinical symptoms) over an extended period of time and have a total or partial control of HIV replication and high CD4+ T-cells counts, others HIV-infected patients show rapid immunological and clinical progression within the first few years after HIV seroconversion (rapid progressors, RPs). This variability in the disease progression in HIV-infected patients is related to a complex interaction among a multitude of factors, including the immune system, genetic background, and viral characteristics among others [3, 4].

Vitamin D (VitD) is an essential nutrient to maintain human health, and its deficiency is related to skeletal diseases (osteomalacia and rickets, among others) and non-skeletal diseases (cancer, diabetes, cardiovascular diseases, autoimmunity, and infectious diseases, among others) [5]. Regarding the immune response against pathogens, VitD triggers antimicrobial pathways in host cells and activates genes that enhance the immunity [6–8]. Hence, the VitD deficiency is related to a higher risk of infection and poor prognosis of infectious diseases such as tuberculosis, influenza, fungal infections, sepsis, and HIV infection [9].

The VitD deficiency is very frequent among HIV-infected patients (around 70–85%) and may be conditioned by HIV-related factors such as ART, HIV infection itself, and higher incidence of malnutrition and comorbidities and non-HIV-related risk factors such as genetic background, advanced age, gender, and limited sunlight exposure [9, 10]. This VitD deficiency has been related to high plasma viral load, increased inflammation and immune activation, decreased CD4+ T-cells, and rapid AIDS progression; whereas higher levels of VitD seem to provide natural resistance to HIV infection [6].

Most of the circulating VitD (85–90%) is tightly bound to vitamin D-binding protein (DBP), also known as GC vitamin D binding protein (GC). Around 10–15% of VitD is less tightly bound to albumin, and only a small fraction of VitD (< 1%) circulates as “free” steroid [11, 12]. VitD has a hydrophobic nature, that binds to DBP with high affinity, particularly calcidiol (25-hydroxycholecalciferol or 25-hydroxyvitamin D; 25(OH)D), which seems to regulate the bioavailability of VitD [13]. The 25(OH)D is the precursor of the active form of VitD, which is converted to the active hormone (1,25-dihydroxycholecalciferol or 1,25-dihydroxyvitamin D; 1,25(OH)2D) in several tissues, including cells of the immune system. Later, the active form of VitD acts on target cells by binding to the vitamin D receptor (VDR), which promotes gene transcription of several target genes and other non-genomics effects [6].

The *DBP* gene has high variability and several single nucleotide polymorphisms (SNPs) in *DBP* gene have been linked to variations in circulating 25(OH)D concentration [14, 15], and chronic diseases such as chronic obstructive pulmonary disease (COPD) and tuberculosis [16, 17]. However, there is scarce information about the influence of *DBP* SNPs on AIDS progression in naïve-HIV-infected patients [18]. Since *DBP* SNPs is associated with VitD levels, it would be plausible that *DBP* SNPs could be related to AIDS progression.

### Objective

We aimed to study the association among *DBP* polymorphisms and the patterns of clinical AIDS progression (LTNPs, MPs, and RPs) in ART-naïve HIV-infected patients.

## Methods

### Patients

This is a retrospective study that was carried out in 667 ART-naïve HIV-infected patients included in two large Spanish cohorts (Cohort of LTNPs and Cohort of the Spanish AIDS Research Network (CoRIS)). Besides, 113 healthy blood donors were used as a Control-group (HIV, hepatitis C virus (HCV), and hepatitis B virus (HBV) negative subjects). Institutional Review Boards of each participating centers approved the programs and all patients signed an informed consent form. This study was conducted under the Declaration of Helsinki and also approved by the Research Ethics Committee of the Instituto de Salud Carlos III (CEI PI_2010-v3).

We classified ART-naïve HIV-infected patients in three groups by their clinical AIDS progression [19, 20]: a) 183 LTNPs patients, who were asymptomatic over 10 years after HIV seroconversion, and with CD4+ ≥500 cells/mm^3^ and RNA-HIV load ≤10,000 copies/ml; b) 334 MPs patients, who had a continuous decrease in CD4+ T-cells (50–100 CD4+/mm^3^ per year) for at least two years after diagnosis of HIV infection; c) 150 RPs patients, who showed two or more values of CD4+ T-cell ≤350 CD4+/mm^3^ and/or who had an AIDS-related event (including death) within three years after HIV seroconversion. HIV-infected patients were without cART during the study period used to stratify by the pattern of AIDS, but patients could be subsequently treated with cART.

After blood extraction, samples were sent to the Spanish HIV HGM Biobank (integrated into the Spanish AIDS Research Network (RIS) and National Netework of Biobanks) and immediately processed and frozen after their reception. The details of the methodology and organization of the Spanish HIV HGM Biobank have been described previously [21, 22]. Afterward, samples from patients were kindly provided by the HIV BioBank for DNA genotyping.

### DNA genotyping

We selected seven *DBP* SNPs (rs16846876, rs12512631, rs2070741, rs2282679, rs7041, rs1155563, rs2298849) previously related to circulating concentrations of VitD metabolites and non-skeletal diseases [17]. These SNPs are located within the coding region (rs7041 [missense variant]), the intronic region (rs2070741, rs2282679, rs1155563 and rs2298849) and the downstream region (rs16846876 and rs12512631) of *DBP* gene.

Total DNA isolation was performed from whole blood with Wizard® SV Genomic DNA Purification System (Promega, Madison, WI, USA). DNA genotyping was performed at the Spanish National Genotyping Center (http://www.cegen.org/ - CeGen) by Agena Bioscience’s MassARRAY platform (San Diego, CA, USA) using the iPLEX® Gold assay design system.

### Statistical analysis

Statistical analyses were performed using the Statistical Package for the Social Sciences (SPSS) 22.0 software (IBM Corp., Chicago, USA) and Stata 15.0 (StataCorp, Texas, USA). All *p*-values were two-tailed and the statistical significance was defined as *p* < 0.05. For the descriptive study, categorical data were analyzed using the chi-squared test or Fisher’s exact test, and continuous variables were analyzed with the Mann-Whitney U and Kruskal-Wallis tests. The genetic association study among *DBP* SNPs and clinical patterns of AIDS progression was evaluated according to dominant, recessive, overdominant, codominant, and additive models by Generalized Linear Models (GLMs) with a binomial distribution (logit-link). Multiple testing correction was performed by the false discovery rate (FDR) with the Benjamini and Hochberg procedure (q-value) in order to exclude spurious associations. Next, the selected SNPs (*q*-value ≤0.05) were valuated by GLMs with a binomial distribution (logit-link) and adjusted by age at the moment of HIV diagnosis, gender, risk group, and *VDR* rs2228570 SNP [23].

Moreover, the Hardy-Weinberg equilibrium (HWE) and pairwise linkage disequilibrium (LD), using the standardized D’ and r^2^ values, were evaluated using Haploview 4.2 software (MIT/Harvard Broad Institute, Cambridge, MA, USA) and haplotype-based association testing was performed using PLINK software (http://zzz.bwh.harvard.edu/plink/index.shtml) by logistic regression.

## Results

### Study population

The characteristics of HIV-infected patients are shown in Table 1. The LTNPs group had the highest values of age at the moment of HIV diagnosis (*p*-value < 0.001) and at the study inclusion (*p*-value < 0.001), and percentage of intravenous drug users (IDU) (*p*-value < 0.001); while LTNPs group had the lowest proportion of HIV-infected male patients (*p*-value < 0.001) and were diagnosed of HIV infection before the year 2000.

**Table 1:** Clinical and epidemiological characteristics of HIV infected patients and healthy donors

	Controls vs. all HIV patients			HIV groups of patients			
Characteristics	Control	All HIV ^(*)^	*p*-value ^(a)^	LTNPs-group	MPs-group	RPs-group	*p*-value^(b)^
No.	113	667		183	334	150	
Male	93 (82.3%)	540 (81.4%)	0.829	115 (64.2%)	283 (84.7%)	142 (94.7%)	**< 0.001**
Age (years)	42.0 (37.0; 49.0)	41.3 (35.0; 48.4)	0.427	48.7 (46.0; 51.7)	38.2 (33.2; 45.3)	38.3 (33.0; 43.8)	**< 0.001**
Age of HIV diagnosis	–	34.3 (29.0; 40.4)	–	39.8 (34.3; 43.7)	31.8 (27.0; 38.4)	34.0 (29.6; 38.1)	**< 0.001**
Year of HIV diagnosis	–	2006 (1999; 2008)	–	1993 (1990; 1997)	2006 (2004; 2008)	2009 (2007; 2010)	**< 0.001**
HIV acquired							
IDU	–	166 (25.0%)	–	130 (72.6%)	29 (8.7%)	7 (4.7%)	**< 0.001**
Homosexual	–	359 (54.1%)	–	13 (7.3%)	220 (65.9%)	126 (84.0%)	
Heterosexual	–	118 (17.8%)	–	27 (15.1%)	76 (22.8%)	15 (10.0%)	
Others	–	20 (3.0%)	–	9 (5.0%)	9 (2.7%)	2 (1.3%)	

Abbreviations: *IDU* Intravenous drug users, *HIV* Human immunodeficiency virus, *LTNPs* Long Term Non Progressors, *MPs* Moderate Progressors, *RPs* Rapid progressors.

Statistics: *P*-values were calculated by Chi-square or Fisher’s exact test, Mann-Whitney and Kruskal-Wallis tests.

^(a)^differences between control group and all HIV infected patients.

^(b)^differences among HIV groups. Statistically significant differences are shown in bold.

^(*)^Clinical and epidemiological data for three HIV-infected patients were not available.

### Characteristics of *DBP* polymorphisms

Additional file 1: Table S1 shows the characteristics of *DBP* polymorphisms in healthy-controls and HIV-infected patientes. All *DBP* SNPs had values for minor allelic frequency (MAF) higher than 5% and the DNA genotyping call-rate success was over 95%. Besides, the genotypic and allelic frequencies of the *DBP* gene were in accordance with the NCBI SNP database for European population (http://www.ncbi.nlm.nih.gov/projects/SNP/). All SNPs were in HWE (*p*-value > 0.05) and had similar genotypic frequencies for *DBP* SNPs in healthy-controls and HIV-infected patients.

**Fig. 1:**
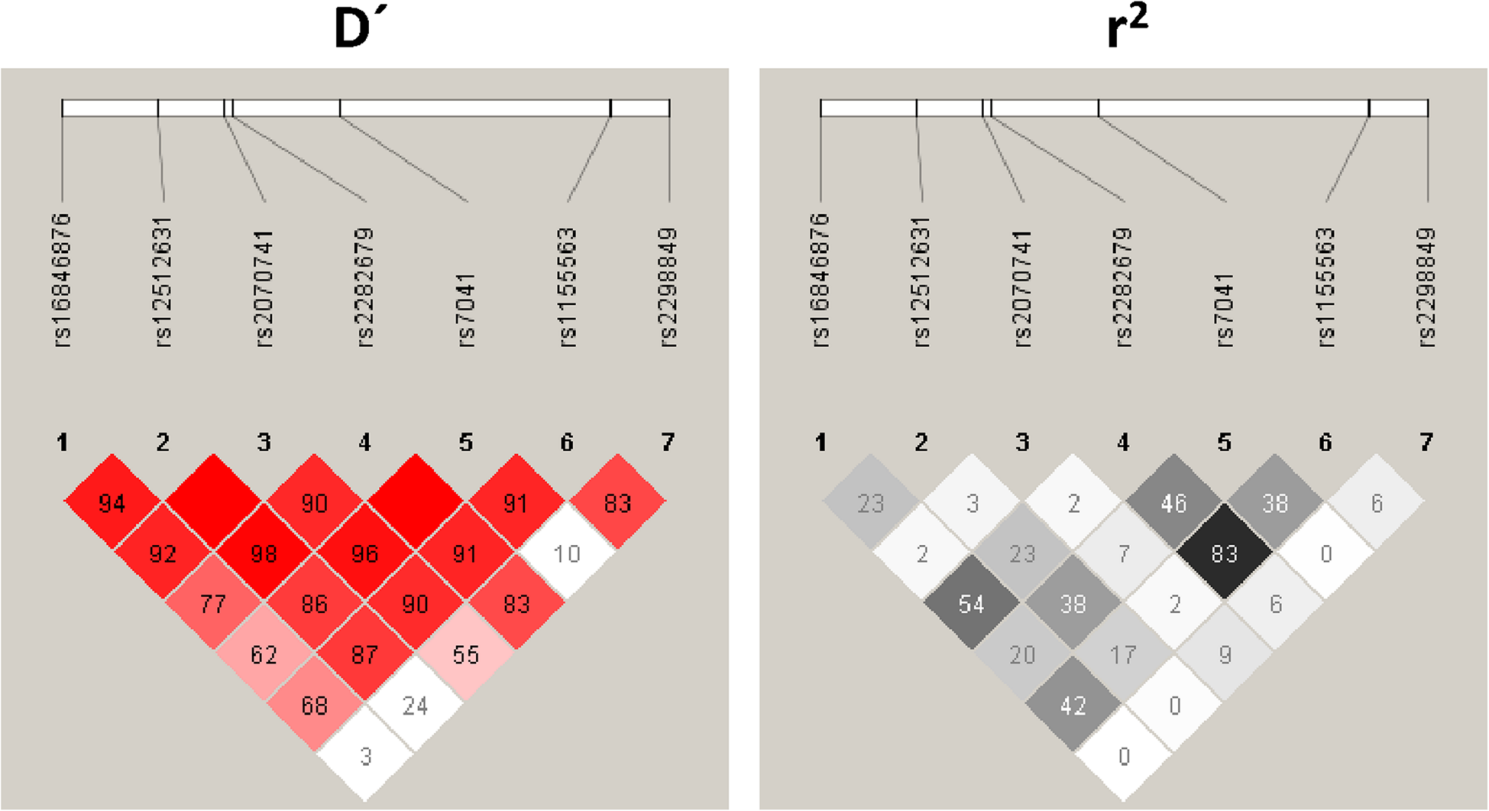
Pairwise linkage disequilibrium (LD) pattern for *DBP* polymorphisms in HIV-infected patients. The grey color intensity decreases with decreasing R-squared value. The location of SNPs is indicated on top. The diagonal represents a SNP and the square represents a pairwise comparison between two SNPs, indicating the magnitude of LD (D’ and r^2^). D’ and r^2^ varies from 0 (absence) to 1 (complete). Abbreviations: DBP, vitamin D binding protein; SNP, single nucleotide polymorphism; LD, linkage disequilibrium; r^2^, square of the correlation coefficient; D’; D-prime or proportion of the possible LD that was present between two SNPs

Figure 1 shows the LD pattern for *DBP* polymorphisms in HIV-infected patients. The LD values were low (D’ < 0.75) for rs2298849 with rs7041 (D’ = 0.10), rs2070741 (D’ = 0.55), rs12512631 (D’ = 0.24), and rs16846876 (D’ = 0.03); and for rs16846876 with rs7041 (D’ = 0.62) and rs1155563 (D’ = 0.68). Besides, the values of r^2^ statistic were low (*r*^2^ < 0.75) among all *DBP* SNPs except for rs2282679 and rs1155563 (*r*^2^ = 0.83), which indicate that most SNPs provide different information.

### *DBP* polymorphisms and AIDS progression

**Fig. 2:**
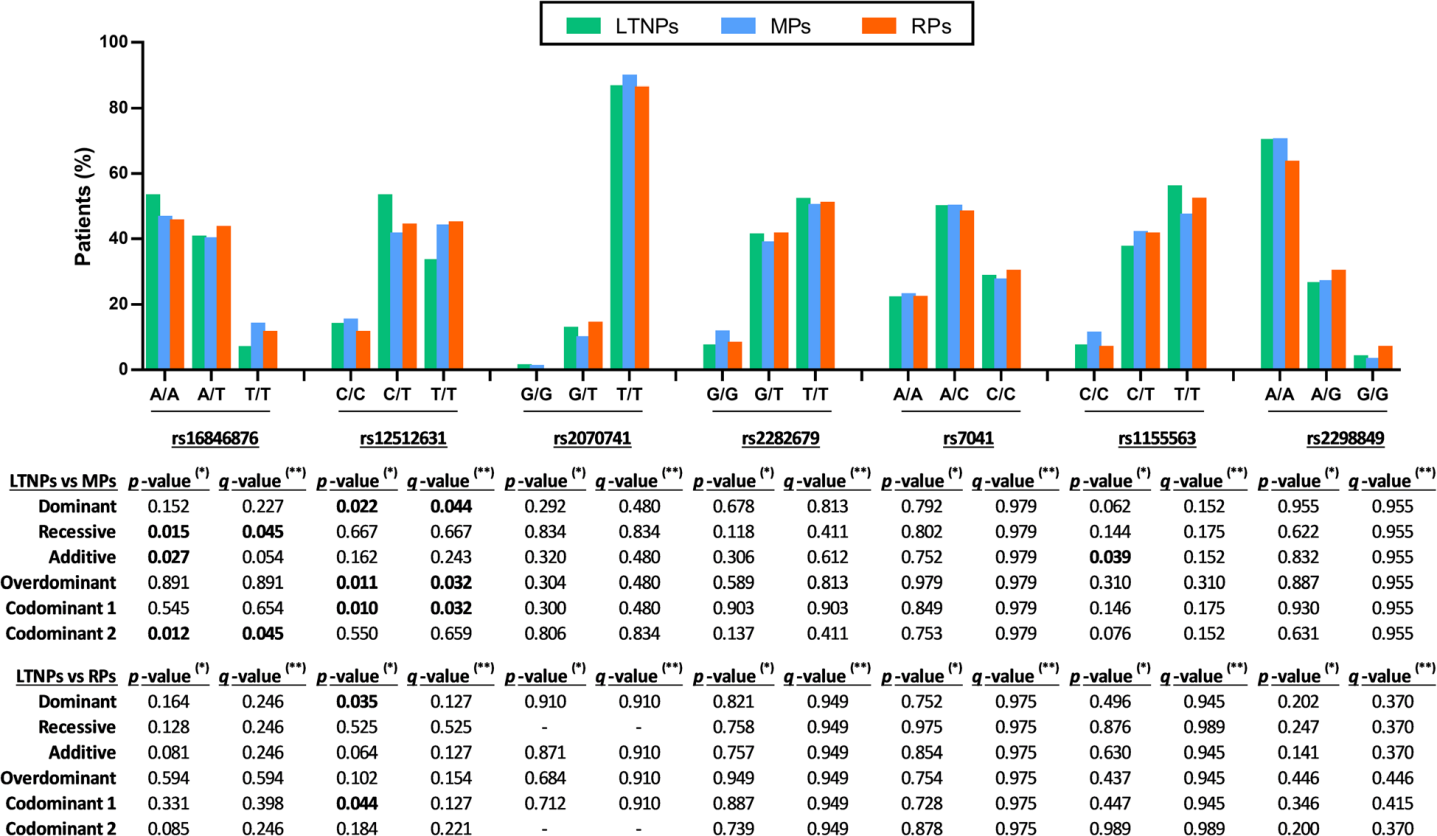
Genetic association of *DBP* polymorphisms with AIDS progression in HIV-infected patients. Statistics: *P*-values were calculated by unadjusted Generalized Linear Models (GLMs) with a binomial distribution (logit-link). (*), raw *p*-values; (**), *p*-values corrected for multiple testing using the false discovery rate (FDR) with Benjamini and Hochberg procedure (*n* = 6 inheritance models, multiple comparisons). Abbreviations: DBP, vitamin D binding protein; LTNPs, Long Term Non Progressors; MPs, Moderate Progressor; RPs, Rapid Progressor; Codominant 1, codominant 1 refers to heterozygous genotype vs more frequent homozygous (genotype 1); Codominant 2, codominant 2 refers to less frequent homozygote (genotype 2) vs more frequent homozygote (genotype 1)

Figure 2 shows the association among *DBP* polymorphisms and AIDS progression by unadjusted GLMs (full description in Additional file 2: Table S2). When comparing LTNPs vs. MPs, rs16846876 showed significant associations with the recessive (*q*-value = 0.045) and codominant (*q*-value = 0.045) models; and rs12512631 showed significant values with the dominant (*q*-value = 0.044), overdominant (*q*-value = 0.032), and codominant (*q*-value = 0.032) models. When comparing LTNPs vs. RPs, we found significant associations for rs12512631 in the dominant (*p*-value = 0.035) and codominant (*p*-value = 0.044) models, but this significant association disappeared after the FDR (Benjamini & Hochberg) controlling procedure. When comparing MPs vs. RPs, there were no significant values.

**Fig. 3:**
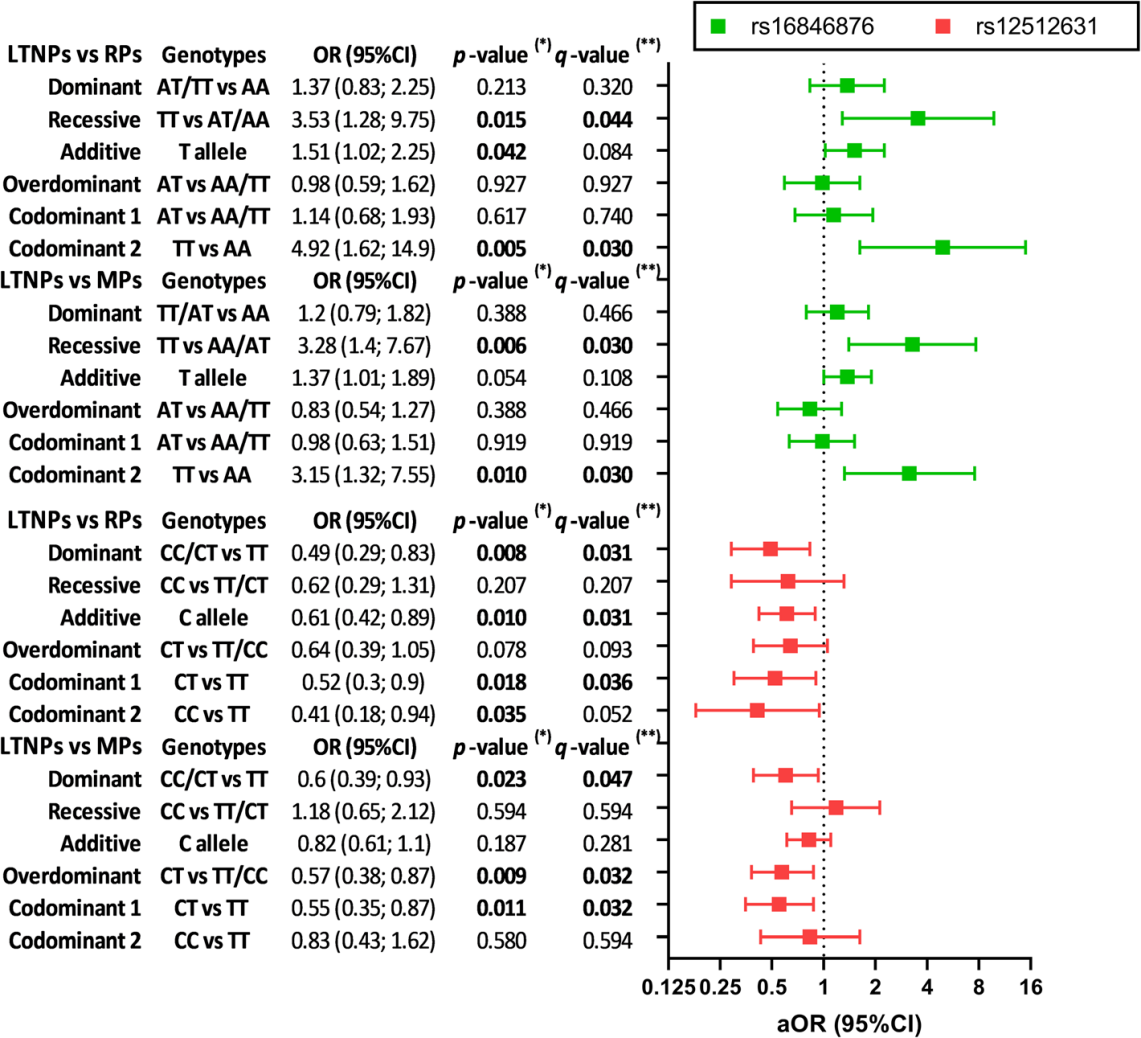
Genetic association of *DBP* polymorphisms with AIDS progression in HIV-infected patients. Statistics: *P*-values were calculated by Generalized Linear Models (GLMs) with a binomial distribution (logit-link) and adjusted for age, gender, and risk category and rs2228570 *VDR* polymorphism. (*), raw *p*-values; (**), *p*-values corrected for multiple testing using the false discovery rate (FDR) with Benjamini and Hochberg procedure (*n* = 6 inheritance models, multiple comparisons). Abbreviations: DBP, vitamin D binding protein; LTNPs, Long Term Non Progressors; MPs, Moderate Progressor; RPs, Rapid Progressor; Codominant 1, codominant 1 refers to heterozygous genotype vs. more frequent homozygous (genotype 1); Codominant 2, codominant 2 refers to less frequent homozygote (genotype 2) vs. more frequent homozygote (genotype 1)

Afterwards, we evaluated the association of rs16846876 and rs12512631 polymorphisms with AIDS progression by GLMs adjusted by age, gender, risk category, and *VDR* rs2228570 SNP (Fig. 3). *DBP* rs16846876 SNP showed significant association under the recessive inheritance model [LTNPs vs. RPs (adjusted odds ratio (aOR) = 3.53; *q*-value = 0.044) and LTNPs vs. MPs (aOR = 3.28; *q*-value = 0.030)] and codominant [LTNPs vs. RPs (aOR = 4.92; *q*-value = 0.030) and LTNPs vs. MPs (aOR = 3.15; *q*-value = 0.030)]. We also found *DBP* rs12512631 SNP showed significant association in the inheritance model dominant [LTNPs vs. RPs (aOR = 0.49; *q*-value = 0.031) and LTNPs vs. MPs (aOR = 0.6; *q*-value = 0.047)], additive [LTNPs vs. RPs (aOR = 0.61; *q*-value = 0.031)], overdominant [LTNPs vs. MPs (aOR = 0.55; *q*-value = 0.032)], and codominant [LTNPs vs RPs (aOR = 0.52; *q*-value = 0.036) and LTNPs vs. MPs (aOR = 0.55; *q*-value = 0.032)].

### *DBP* haplotypes and AIDS progression

Table 2 shows the adjusted association of *DBP* haplotypes (composed by rs16846876 and rs12512631) with the patterns of AIDS progression. When comparing LTNPs vs. RPs, we found significant associations for the *DBP* haplotype AC (aOR = 0.63; *q*-value = 0.028) and the *DBP* haplotype TT (aOR = 1.64; *q*-value = 0.028).

**Table 2:** Distribution of *vitamin D binding protein (DBP)* haplotypes (composed by rs16846876 and rs12512631) and its association with the patterns of clinical AIDS progression (LTNPs, MPs, RPs) in HIV infected patients

		Univariate			Multivariate		
Haplotypes	Freq.	aOR (95CI)	*p*-value ^(*)^	*q*-value ^(**)^	aOR (95CI)	p-value ^(*)^	q-value ^(**)^
LTNPs vs MPs							
AC	0.366	0.85 (0.65; 1.10)	0.218	0.284	0.85 (0.63; 1.15)	0.292	0.378
TT	0.307	1.45 (1.09; 1.93)	**0.009**	**0.027**	2.32 (4.95; 20.8)	**0.018**	0.054
AT	0.321	0.86 (0.66; 1.13)	0.284	0.284	0.87 (0.65; 1.18)	0.378	0.378
LTNPs vs RPs							
AC	0.361	0.74 (0.53; 1.04)	0.080	0.120	0.63 (0.43; 0.93)	**0.019**	**0.028**
TT	0.286	1.43 (1.01; 2.02)	**0.044**	0.120	1.64 (1.09; 2.46)	**0.017**	**0.028**
AT	0.343	0.99 (0.73; 1.37)	0.973	0.973	1.07 (0.74; 1.54)	0.708	0.708

Abbreviations: *aOR* Adjusted odds ratio, *95 CI* 95% confidence interval, *HIV* Human immunodeficiency virus, *DBP* Vitamin D binding protein, *LTNPs* Long term non progressors, *MPs* moderate progressor, *RPs* Rapid progressor.

Statistics: *P*-values were calculated using PLINK software by logistic regression adjusted by gender, age at HIV diagnosis, men who had sex with men and *VDR* rs2228570 polymorphism.

^(*)^raw *p*-values

^(**)^*p*-values corrected for multiple testing (q-value) using the false discovery rate (FDR) with Benjamini and Hochberg procedure (*n* = 3 haplotypes, multiple comparisons). Statistically significant differences are shown in bold.

## Discussion

In this study, rs16846876 and rs12512631 SNPs were associated with progression of HIV infection (LTNPs vs. MPs) and progression to AIDS (LTNPs vs. RPs) in ART-naïve HIV-infected patients from two Spanish large cohorts (cohorts of LTNP and CoRIS), which collected patients from all over Spain. Besides, this last association (LTNPs vs. RPs) was confirmed in the analysis of *DBP* haplotypes (composed by rs16846876 and rs12512631).

Our study was performed in HIV-infected patients came from all different regions of Spain. This detail is an interesting plus of our study because the genetic diversity that could be found in Spain is better represented. Besides, a variability in sun exposure is also to be expected since Spain has characteristics of seasonality and radiation that vary between regions, which may impact in the conversion rate of pro-vitamin D to pre-vitamin D.

The *DBP* gene is highly polymorphic, and some SNPs have been linked to VitD concentration in serum of general population [17] and HIV infected patients [24]. *DBP* SNPs are associated with the delivery of 1,25(OH)2D to target tissues, as well as the removal of VitD metabolites from circulation [14–16]. These *DBP* variants may modulate protein expression or its activity and, thus, may affect synthesis, distribution, and function of VitD. Two of the SNPs more studied are rs7041 and rs4588 polymorphisms, which are missense variants that produce different isoforms of the DBP protein (D (Asp) > E (Glu) and T (Thr) > M (Met), respectively) with different affinity for VitD metabolites [25, 26]. These isoforms seem to affect the delivery of VitD at the cellular levels or tissues [16]. The analysis of HapMap data shows that there are a very high number of SNPs that are in LD with rs7041 and rs4588, and therefore other SNPs could also be related to this effect [18]. Besides, other *DBP* SNPs, as those analyzed in this study (rs16846876, rs12512631, rs2070741, rs2282679, rs1155563, and rs2298849), have already been related to variations in circulating 25(OH)D concentration [17]. Furthermore, some of these SNPs have been related to osteoporosis, cancer, diabetes, neurodegenerative disorders, autoimmunity, asthma, COPD, and tuberculosis [17, 18]. Regarding HIV infection, there are scarce reports about *DBP* SNPs and AIDS progression in naïve-HIV-infected patients, whose have found contradictory results [18]. Several articles did not find any significant association between *DBP* SNPs and the clinical progression of HIV infection [27–33]; while only one report found a significant association between *DBP* SNPs and AIDS progression in naïve-HIV-infected patients [34], which coincide with our findings.

In our study, we described a significant association between rs16846876 and rs12512631 SNPs and clinical progression of HIV infection. Regarding the possible action mechanism of the studied polymorphisms, the *DBP* rs12512631 polymorphism has been linked to the methylation status of the *DBP* gene, possibly affecting gene transcription and phenotypic characteristics [35]. In the literature, several studies found an association between the rs12512631 polymorphism and serum 25(OH)D concentration in the healthy population [36] and in the cancer patients [16, 35]. Besides, differences in the strength of the association between rs12512631 and 25(OH)D concentration seem to vary according to the patient characteristics [37]. In this setting, Miettinen et al. [37] reported a significant association of rs12512631 with 25(OH)D concentration in the mothers of type 1 diabetic children, while there was no association in the mothers of non-diabetic children during pregnancy. Additionally, rs12512631 was associated with 25(OH)D levels in different ways in young people and adults [37]. In these studies, rs12512631 C allele was associated with high concentration of VitD [36, 37], which conceptually agrees with our data. The presence of C allele was associated with protection against progression of HIV infection (LTNPs vs. MPs) and progression to AIDS (LTNPs vs. RPs), possibly because the plasma VitD concentration in these patients was higher. The *DBP* rs16846876 polymorphism has been also linked to serum 25(OH) D concentrations in healthy subjects [36], pregnant women [38], and cancer patients [16]. In the literature, rs16846876 TT genotype was associated with lower concentration of VitD [36, 38], which is in acordance with our results. The presence of TT genotype was associated with higher odds of progression of HIV infection (LTNPs vs. MPs), possibly because the plasma VitD concentration was lower than in presence of AA and AT genotypes. Little is known about *DBP* rs16846876 polymorphism and its possible role on gene expression or another molecular mechanism. By using HaploReg v4.1 software (https://pubs.broadinstitute.org/mammals/haploreg/haploreg.php), which is a bioinformatic tool for exploring candidate regulatory SNPs on haplotype blocks, we found that *DBP* rs16846876 polymorphism is in high LD with several SNPs such as rs11732044, rs12648331, rs56003670, rs60696209 and rs2201124, which are involved in histone modifications with enhancer properties. Histone modifications can alter chromatine structure, which determines the DNA accessibility. The actively transcribed regions are usually located in looser chromatin regions, so RNA polymerases and transcription factors can access more easily. In this context, the observed role of the rs16846876 polymorphism on the HIV progression could be related with epigenetic changes in an indirect way, reflecting the regulation of *DBP* expression caused by SNPs in high LD with rs16846876 polymorphism.

Moreover, in the current study, *DBP* haplotypes (comprised of rs16846876 and rs12512631) were also investigated to analyze the association with HIV progression. Patients with TT haplotype (unfavorable alleles) had two twice more odds of HIV progression, while patients AC haplotype (favorable alleles) had decreased odds. These associations confirm the previous findings for individual SNPs. However, the results obtained with the analysis of haplotypes did not improve those obtained for individual SNPs, where we found that patients carrying the rs16846876 TT genotype had a probability of around four times more HIV progression than patients carrying AA or AT genotype.

### Limitations of the study

Firstly, the limited number of patients per group that may have decreased the statistical power and the number of significant values, particularly after FDR controlling multiple testing. Secondly, the differences between the three groups of patients (LTNPs, MPs, and RPs) in demographic and clinical characteristics were substantial and they could have introduced some bias in the study, although we accounted for these variables in the statistical analysis. This is due to the idiosyncrasy of each group. Thirdly, we did not have data of DBP and VitD in the plasma because the adequate sample was not available in most patients. Besides, non-AIDS-related comorbidities were not available, particularly information about liver disease which directly affects to DBP levels in the body.

## Conclusions

*DBP* rs16846876 and rs12512631 SNPs are related to the patterns of clinical AIDS progression (LTNPs, MPs, and RPs) in ART-naïve HIV-infected patients. Our findings provide new knowledge about AIDS progression that may be relevant to a better understanding of the pathogenesis of HIV infection.

### Supplementary information

**Additional file 1: Table S1.** Characteristics of *vitamin D binding protein (DBP)* polymorphisms in HIV infected patients and healthy donors.

**Additional file 2: Table S2.** Genetic association of *vitamin D binding protein (DBP)* polymorphisms with distinct patterns of AIDS progression in HIV infected patients.

## Data Availability

The datasets analyzed during the current study may be available upon reasonable request.

## Appendix 1

### Annex: Centers and investigators involved in CoRIS

**Executive committee**: Santiago Moreno, Julia del Amo, David Dalmau, Maria Luisa Navarro, Maria Isabel González, Jose Luis Blanco, Federico Garcia, Rafael Rubio, Jose Antonio Iribarren, Félix Gutiérrez, Francesc Vidal, Juan Berenguer, Juan González.

**Fieldwork, data management and analysis**: Julia Del Amo, Inma Jarrín, Belén Alejos, Victoria Hernando, Cristina Moreno, Carlos Iniesta, Luis Miguel Garcia Sousa, Nieves Sanz Perez.

**BioBanK HIV:** Hospital General Universitario Gregorio Marañón: M Ángeles Muñoz-Fernández, Isabel María García-Merino, Irene Consuegra Fernández, Coral Gómez Rico, Jorge Gallego de la Fuente, Paula Palau Concejo.

**Participating centres:**

Hospital General Universitario de Alicante (Alicante): Joaquín Portilla, Esperanza Merino, Sergio Reus, Vicente Boix, Livia Giner, Carmen Gadea, Irene Portilla, María Pampliega, Marcos Díez, Juan Carlos Rodríguez, José Sánchez-Payá.

**Hospital Universitario de Canarias (San Cristobal de la Laguna):** Juan Luis Gómez, Jehovana Hernández, María Remedios Alemán, María del Mar Alonso, María Inmaculada Hernández, Felicitas Díaz-Flores, Dácil García, Ricardo Pelazas., Ana López Lirola.

**Hospital Universitario Central de Asturias (Oviedo):** José Sanz Moreno, Alberto Arranz Caso, Cristina Hernández Gutiérrez, María Novella Mena.

**Hospital Universitario 12 de Octubre (Madrid):** Rafael Rubio, Federico Pulido, Otilia Bisbal, Asunción Hernando, Lourdes Domínguez, David Rial Crestelo, Laura Bermejo, Mireia Santacreu.

**Hospital Universitario de Donostia (Donostia-San Sebastián):** José Antonio Iribarren, Julio Arrizabalaga, María José Aramburu, Xabier Camino, Francisco Rodríguez-Arrondo, Miguel Ángel von Wichmann, Lidia Pascual Tomé, Miguel Ángel Goenaga, Mª Jesús Bustinduy, Harkaitz Azkune, Maialen Ibarguren, Aitziber Lizardi, Xabier Kortajarena.

**Hospital General Universitario De Elche (Elche):** Félix Gutiérrez, Mar Masiá, Sergio Padilla, Andrés Navarro, Fernando Montolio, Catalina Robledano, Joan Gregori Colomé, Araceli Adsuar, Rafael Pascual, Marta Fernández, Elena García, José Alberto García, Xavier Barber.

**Hospital General Universitario Gregorio Marañón (Madrid):** Juan Berenguer, Juan Carlos López Bernaldo de Quirós, Isabel Gutiérrez, Margarita Ramírez, Belén Padilla, Paloma Gijón, Teresa Aldamiz-Echevarría, Francisco Tejerina, Francisco José Parras, Pascual Balsalobre, Cristina Diez, Leire Pérez Latorre.

**Hospital Universitari de Tarragona Joan XXIII (Tarragona):** Francesc Vidal, Joaquín Peraire, Consuelo Viladés, Sergio Veloso, Montserrat Vargas, Miguel López-Dupla, Montserrat Olona, Anna Rull, Esther Rodríguez-Gallego, Verónica Alba.

**Hospital Universitario y Politécnico de La Fe (Valencia):** Marta Montero Alonso, José López Aldeguer, Marino Blanes Juliá, María Tasias Pitarch, Iván Castro Hernández, Eva Calabuig Muñoz, Sandra Cuéllar Tovar, Miguel Salavert Lletí, Juan Fernández Navarro.

**Hospital Universitario La Paz/IdiPAZ:** Juan González-garcia, Francisco Arnalich, José Ramón Arribas, Jose Ignacio Bernardino de la Serna, Juan Miguel Castro, Luis Escosa, Pedro Herranz, Victor Hontañón, Silvia García-Bujalance, Milagros García López-Hortelano, Alicia González-Baeza, Maria Luz Martín-Carbonero, Mario Mayoral, Maria Jose Mellado, Rafael Esteban Micán, Rocio Montejano, María Luisa Montes, Victoria Moreno, Ignacio Pérez-Valero, Berta Rodés, Talia Sainz, Elena Sendagorta, Natalia Stella Alcáriz, Eulalia Valencia.

**Hospital San Pedro Centro de Investigación Biomédica de La Rioja (CIBIR) (Logroño):** José Ramón Blanco, José Antonio Oteo, Valvanera Ibarra, Luis Metola, Mercedes Sanz, Laura Pérez-Martínez.

**Hospital Universitari MutuaTerrassa (Terrasa):** David Dalmau, Angels Jaén, Montse Sanmartí, Mireia Cairó, Javier Martinez-Lacasa, Pablo Velli, Roser Font, Mariona Xercavins, Noemí Alonso.

**Complejo Hospitalario de Navarra (Pamplona):** María Rivero, Jesús Repáraz, María Gracia Ruiz de Alda, María Teresa de León Cano, Beatriz Pierola Ruiz de Galarreta.

**Corporació Sanitària Parc Taulí (Sabadell):** Ferrán Segura, María José Amengual, Gemma Navarro, Montserrat Sala, Manuel Cervantes, Valentín Pineda, Sonia Calzado, Marta Navarro.

**Hospital Universitario de La Princesa (Madrid):** Ignacio de los Santos, Jesús Sanz Sanz, Ana Salas Aparicio, Cristina Sarriá Cepeda, Lucio Garcia-Fraile Fraile, Enrique Martín Gayo.

**Hospital Universitario Ramón y Cajal (Madrid):** Santiago Moreno, José Luis Casado, Fernando Dronda, Ana Moreno, María Jesús Pérez Elías, Cristina Gómez Ayerbe, Carolina Gutiérrez, Nadia Madrid, Santos del Campo Terrón, Paloma Martí, Uxua Ansa, Sergio Serrano, María Jesús Vivancos.

**Hospital General Universitario Reina Sofía (Murcia):** Enrique Bernal, Alfredo Cano, Antonia Alcaraz García, Joaquín Bravo Urbieta, Ángeles Muñoz, Maria Jose Alcaraz, Maria del Carmen Villalba.

**Hospital Nuevo San Cecilio (Granada):** Federico García, José Hernández, Alejandro Peña, Leopoldo Muñoz, Paz Casas, Marta Alvarez, Natalia Chueca, David Vinuesa, Clara Martinez-Montes.

**Centro Sanitario Sandoval (Madrid):** Jorge Del Romero, Carmen Rodríguez, Teresa Puerta, Juan Carlos Carrió, Mar Vera, Juan Ballesteros, Oskar Ayerdi.

**Hospital Universitario Son Espases (Palma de Mallorca):** Melchor Riera, María Peñaranda, María Leyes, Mª Angels Ribas, Antoni A Campins, Carmen Vidal, Francisco Fanjul, Javier Murillas, Francisco Homar.

**Hospital Universitario Virgen de la Victoria (Málaga):** Jesús Santos, Crisitina Gómez Ayerbe, Isabel Viciana, Rosario Palacios, Carmen María González.

**Hospital Universitario Virgen del Rocío (Sevilla):** Pompeyo Viciana, Nuria Espinosa, Luis Fernando López-Cortés.

**Hospital Universitario de Bellvitge (Hospitalet de Llobregat):** Daniel Podzamczer, Elena Ferrer, Arkaitz Imaz, Juan Tiraboschi, Ana Silva, María Saumoy.

**Hospital Costa del Sol (Marbella):** Julián Olalla, Alfonso del Arco, Javier de la torre, José Luis Prada, José María García de Lomas Guerrero, Javier Pérez Stachowski.

**Hospital General Universitario Santa Lucía (Cartagena):** Onofre Juan Martínez, Francisco Jesús Vera, Lorena Martínez, Josefina García, Begoña Alcaraz, Amaya Jimeno.

**Complejo Hospitalario Universitario a Coruña (Chuac) (A Coruña):** Angeles Castro Iglesias, Berta Pernas Souto, Alvaro Mena de Cea**.**

**Hospital Universitario Virgen de la Arrixaca (El Palmar):** Carlos Galera, Helena Albendin, Aurora Pérez, Asunción Iborra, Antonio Moreno, Maria Angustias Merlos, Asunción Vidal.

**Hospital Universitario Infanta Sofia (San Sebastian de los Reyes):** Inés Suárez-García, Eduardo Malmierca, Patricia González-Ruano, Dolores Martín Rodrigo.

**Complejo Hospitalario de Jaén (Jaén):** Mohamed Omar Mohamed-Balghata, María Amparo Gómez Vidal.

**Hospital Clínico San Carlos (Madrid):** Vicente Estrada Pérez, Maria Jesus Téllez Molina, Jorge Vergas García, Juncal Pérez-Somarriba Moreno.

**Hospital Universitario Fundación Jiménez Díaz (Madrid):** Miguel Górgolas., Alfonso Cabello., Beatriz Álvarez., Laura Prieto.

**Hospital Universitario Príncipe de Asturias (Alcalá de Henares):** José Sanz Moreno, Alberto Arranz Caso, Cristina Hernández Gutiérrez, María Novella Mena.

**Hospital Clínico Universitario de Valencia (València):** María José Galindo Puerto, Ramón Fernando Vilalta, Ana Ferrer Ribera.

**Hospital Reina Sofía (Córdoba):** Antonio Rivero Román, Maria Teresa Brieva Herrero, Antonio Rivero Juárez, Pedro López López, Isabel Machuca Sánchez, José Peña Martínez.

**Hospital Universitario Severo Ochoa (Leganés):** Miguel Cervero Jiménez, Rafael Torres Perea, Juan José Jusdado Ruiz-Capillas.

**Nuestra Señora de Valme:** Juan A Pineda.

## Appendix 2

**Centers involved in Long Term Non-Progressors (LTNP) chort:**

C. Sandoval - Madrid.

H. 12 de Octubre - Madrid.

H. Arnau de Vilanova - Lleida.

H. Asturias.

H. Bellvitge - Barcelona.

H. Castellón.

H. Clínic - Barcelona.

H. Donostia - San Sebastián.

H. Elche - Alicante.

H. Germans Trias i Pujol - Badalona.

H. Gregorio Marañón - Madrid.

H. Joan XXIII - Tarragona.

H. La Fe - Valencia.

H. La Paz/Carlos III - Madrid.

H. La Princesa - Madrid.

H. Navarra - Pamplona.

H. Parc Taulí- Sabadell.

H. Ramón y Cajal - Madrid.

H. San Cecilio - Granada.

H. San Pedro - Logroño.

H. Son Dureta - Mallorca.

H. Virgen del Rocío – Sevilla

## Abbreviations

1,25-(OH)2D: 1,25-dihydroxyvitamin D
25(OH)D: 25-hydroxyvitamin D
AIDS: Acquired immunodeficiency syndrome
aOR: Adjusted odds ratio
ART: Antiretroviral treatment
COPD: Chronic obstructive pulmonary disease
CoRIS: Cohort of the Spanish AIDS Research Network
DBP: Vitamin D-binding protein
FDR: False discovery rate
GC: GC vitamin D binding protein
GLMs: Generalized Linear Models
HIV: Human immunodeficiency virus
HWE: Hardy-Weinberg equilibrium
IDU: Intravenous drug users
LD: Linkage disequilibrium
LTNPs: Long-term non-progressors
MPs: Moderate progressors
RPs: Rapid progressors
SNPs: Single nucleotide polymorphisms
SPSS: Statistical Package for the Social Sciences
ST: Supplementary Table
VDR: Vitamin D receptor
VitD: Vitamin D
