# Supplementary material for: DBP rs16846876 and rs12512631 polymorphisms are associated with progression to AIDS naïve HIV-infected patients: a retrospective study
Source: J Biomed Sci. 2019 Oct 23;26:83. doi: 10.1186/s12929-019-0577-y (PMC6806573; doi:10.1186/s12929-019-0577-y)
Supplement: Supplementary file 1 — Additional file 1: Table S1. Characteristics of vitamin D binding protein (DBP) polymorphisms in HIV infected patients and healthy donors. [file 12929_2019_577_MOESM1_ESM.docx]

**Table 1.** Clinical and epidemiological characteristics of HIV infected patients and healthy donors.

|  | **Controls vs. all HIV patients** | | | **HIV groups of patients** | | | |
| --- | --- | --- | --- | --- | --- | --- | --- |
| **Characteristics** | **Control** | **All HIV ^(*)^** | **p-value ^(a)^** | **LTNPs-group** | **MPs-group** | **RPs-group** | ***p*-value^(b)^** |
| **No.** | 113 | 667 |  | 183 | 334 | 150 |  |
| **Male** | 93 (82.3%) | 540 (81.4%) | 0.829 | 115 (64.2%) | 283 (84.7%) | 142 (94.7%) | **<0.001** |
| **Age (years)** | 42.0 (37.0; 49.0) | 41.3 (35.0; 48.4) | 0.427 | 48.7 (46.0; 51.7) | 38.2 (33.2; 45.3) | 38.3 (33.0; 43.8) | **<0.001** |
| **Age of HIV diagnosis** | - | 34.3 (29.0; 40.4) | - | 39.8 (34.3; 43.7) | 31.8 (27.0; 38.4) | 34.0 (29.6; 38.1) | **<0.001** |
| **Year of HIV diagnosis** | - | 2006 (1999; 2008) | - | 1993 (1990; 1997) | 2006 (2004; 2008) | 2009 (2007; 2010) | **<0.001** |
| **HIV acquired** |  |  |  |  |  |  |  |
| **IDU** | - | 166 (25.0%) | - | 130 (72.6%) | 29 (8.7%) | 7 (4.7%) | **<0.001** |
| **Homosexual** | - | 359 (54.1%) | - | 13 (7.3%) | 220 (65.9%) | 126 (84.0%) |  |
| **Heterosexual** | - | 118 (17.8%) | - | 27 (15.1%) | 76 (22.8%) | 15 (10.0%) |  |
| **Others** | - | 20 (3.0%) | - | 9 (5.0%) | 9 (2.7%) | 2 (1.3%) |  |

**Statistics**: P-values were calculated by Chi-square or Fisher’s exact test, Mann-Whitney and Kruskal-Wallis tests: (a). differences between control group and all HIV infected patients; (b). differences among HIV groups. Statistically significant differences are shown in bold.

(*), Clinical and epidemiological data for three HIV-infected patients were not available.

**Abbreviations**: IDU, intravenous drug users; HIV, Human immunodeficiency virus; LTNPs, Long Term Non Progressors; MPs, Moderate Progressors; RPs, Rapid progressors.
