# Supplementary material for: DBP rs16846876 and rs12512631 polymorphisms are associated with progression to AIDS naïve HIV-infected patients: a retrospective study
Source: J Biomed Sci. 2019 Oct 23;26:83. doi: 10.1186/s12929-019-0577-y (PMC6806573; doi:10.1186/s12929-019-0577-y)
Supplement: Supplementary file 2 — Additional file 2: Table S2. Genetic association of vitamin D binding protein (DBP) polymorphisms with distinct patterns of AIDS progression in HIV infected patients. [file 12929_2019_577_MOESM2_ESM.docx]

**Table 2.** Distribution of *vitamin D binding protein (DBP)* haplotypes (composed by rs16846876 and rs12512631) and its association with the patterns of clinical AIDS progression (LTNPs, MPs, RPs) in HIV infected patients.

|  |  | **Univariate** | | | **Multivariate** | | |
| --- | --- | --- | --- | --- | --- | --- | --- |
| **Haplotypes** | **Freq.** | **aOR (95CI)** | ***p*-value (*)** | ***q*-value (**)** | **aOR (95CI)** | **p-value (*)** | **q-value (**)** |
| LTNPs vs MPs |  |  |  |  |  |  |  |
| AC | 0.366 | 0.85 (0.65; 1.10) | 0.218 | 0.284 | 0.85 (0.63; 1.15) | 0.292 | 0.378 |
| TT | 0.307 | 1.45 (1.09; 1.93) | **0.009** | **0.027** | 2.32 (4.95; 20.8) | **0.018** | 0.054 |
| AT | 0.321 | 0.86 (0.66; 1.13) | 0.284 | 0.284 | 0.87 (0.65; 1.18) | 0.378 | 0.378 |
| LTNPs vs RPs |  |  |  |  |  |  |  |
| AC | 0.361 | 0.74 (0.53; 1.04) | 0.080 | 0.120 | 0.63 (0.43; 0.93) | **0.019** | **0.028** |
| TT | 0.286 | 1.43 (1.01; 2.02) | **0.044** | 0.120 | 1.64 (1.09; 2.46) | **0.017** | **0.028** |
| AT | 0.343 | 0.99 (0.73; 1.37) | 0.973 | 0.973 | 1.07 (0.74; 1.54) | 0.708 | 0.708 |

**Statistics**: P-values were calculated using PLINK software by logistic regression adjusted by gender, age at HIV diagnosis, men who had sex with men and *VDR* rs2228570 polymorphism. (*), raw p-values; (**), p-values corrected for multiple testing (q-value) using the false discovery rate (FDR) with Benjamini and Hochberg procedure (n= 3 haplotypes, multiple comparisons). Statistically significant differences are shown in bold.

**Abbreviations**: aOR, adjusted odds ratio; 95 CI, 95 of confidence interval; HIV, human immunodeficiency virus; DBP, vitamin D binding protein; LTNPs, long term non progressors; MPs, moderate progressor; RPs, rapid progressor.
